# Bis(isonicotinamide-κ*N*)silver(I) tri­fluoro­methane­sulfonate aceto­nitrile disolvate

**DOI:** 10.1107/S2414314621010737

**Published:** 2021-10-21

**Authors:** Rafael A. Adrian, Sara J. Ibarra, Hadi D. Arman

**Affiliations:** a Department of Chemistry and Biochemistry, University of the Incarnate Word, San Antonio Texas 78209, USA; b Department of Chemistry, The University of Texas at San Antonio, San Antonio Texas 78249, USA; University of Antofagasta, Chile

**Keywords:** crystal structure, silver atom, isonicotinamide, tri­fluoro­methane­sulfonate ions, aceto­nitrile, polymeric structure

## Abstract

The central Ag^I^ atom of the title salt, [Ag(INAM)_2_](CF_3_SO_3_)·2CH_3_CN, where INAM is isonicotinamide (C_6_H_6_N_2_O), is twofold coordinated by the pyridine N atoms of two isonicotinamide ligands creating a slightly distorted linear mol­ecular geometry. The formation of polymeric chains {[Ag(INAM)_2_]^+^}_*n*_, held together by discrete hydrogen bonds through the amide group of the INAM ligand leaves voids for non-coordinating aceto­nitrile mol­ecules that inter­act the silver metal center *via* regium bonds.

## Abstract 2

The reaction of silver(I) triflouro­methane­sulfonate with isonicotinamide in aceto­nitrile produces a polymeric structure held together by discrete hydrogen bonds, regium bonds between the metal atom and the solvent mol­ecules, and metal–metal inter­actions.

## Graphical abstract

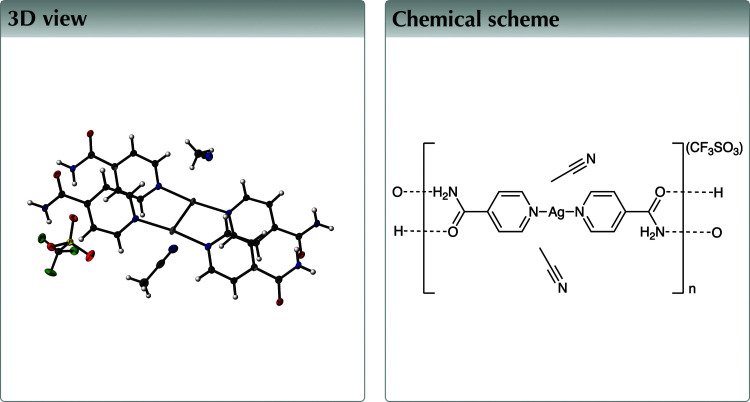

## Structure description

Silver(I) isonicotinamide complexes have been investigated for the ability to form coordination complexes with a variety of mol­ecular geometries due to amide hydrogen-bond synthons in their structure (Aakeröy & Beatty, 1998 ▸; Aakeröy *et al.*, 1998 ▸; Lian *et al.* 2007 ▸), luminescent properties (Yeşilel *et al.*, 2012 ▸), and anti­bacterial activity (Abu-Youssef *et al.*, 2007 ▸; Yu *et al.*, 2020 ▸). Our research group inter­est currently lies in the synthesis of novel metal complexes with biological activity; as part of our research in this area, herein, we describe the synthesis and structure of the title silver(I) complex.

**Figure 1:**
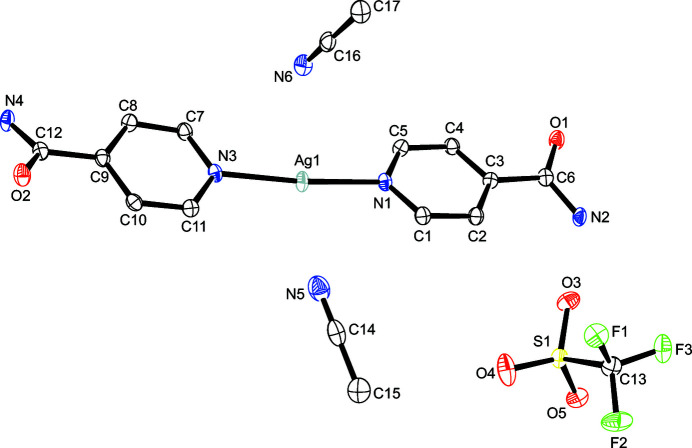
The mol­ecular structure of the title compound with displacement ellipsoids drawn at the 50% probability level; H atoms are omitted for clarity.

As depicted in Fig. 1 ▸, the asymmetric unit of the title compound shows the Ag^I^ ion in a distorted linear coordination environment defined by two N-bonded isonicotamide ligands. Two aceto­nitrile mol­ecules and a tri­fluoro­methane­sulfonate ion complete the asymmetric unit; the aceto­nitrile mol­ecules sit at opposite sides of the plane defined by N1—Ag1—N3 with the nitrile group facing the silver(I) metal center. All relevant bond lengths and angles involving the Ag atom are presented in Table 1 ▸. The angle N1—Ag1—N3 of 172.78 (7) is within the reported values (174.9, 180, and 171.1) in the comparable silver(I) isonicotinamide structures currently available in the CSD (version 5.42 with update May 2021; Aakeröy & Beatty, 1998 ▸; refcode NISNEI; Bhogala *et al.*, 2004 ▸; refcode NABYOF; Abu-Youssef *et al.*, 2007 ▸; refcode XECZUB01).

**Figure 2:**
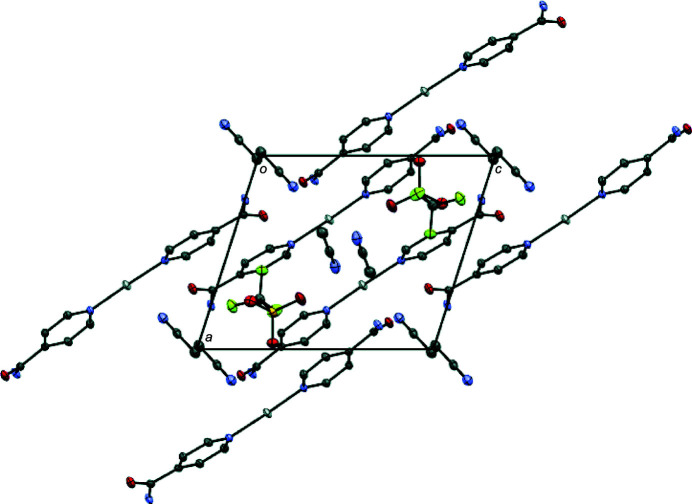
Perspective view of the packing structure of the title complex along the crystallographic *b* axis; H atoms are omitted for clarity.

Two types of hydrogen-bonding motifs are present in the crystal lattice, with numerical values collated in Table 2 ▸. In the crystal packing, mol­ecules self-assemble into layers aligned along the *a-*axis direction (Fig. 2 ▸) *via* N—H⋯O inter­actions. The tri­fluoro­methane­sulfonate anions fill the void between the layers and inter­act with the isonicotinamide ligands through additional N—H⋯O inter­actions. The pyridyl rings of the isonicotinamide ligand show π–π stacking inter­actions with centroid-to-centroid (*Cg*⋯*Cg*) distances ranging from 3.7005 (13) to 3.8503 (14) Å, and offset distances ranging from 1.940 to 2.056 Å, respectively.

**Figure 3:**
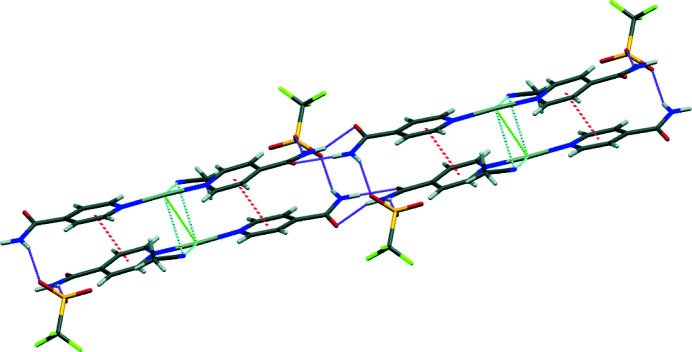
Capped sticks representation of the title mol­ecule showing hydrogen bonds inter­actions (violet), regium bonds (light blue), Ag⋯Ag inter­actions (light green), and π–π stacking inter­actions (red).

Two different supra­molecular inter­actions involving the silver atom are also responsible for the observed crystal packing: an Ag⋯Ag inter­action with a distance between silver atoms of 3.4258 (3) Å, comparable to other silver complexes found in the CSD database (Titov *et al.*, 2018 ▸; refcode FINWOR; Titov *et al.*, 2019 ▸; refcode PIRCUR); and regium bonds, between the nitro­gen of the aceto­nitrile solvent mol­ecules and the silver atom (Alkorta *et al.*, 2020 ▸; Zierkiewicz *et al.*, 2018 ▸), with lengths of 2.916 Å for Ag1—N5 and 2.955 Å for Ag1—N6. (Fig. 3 ▸)

## Synthesis and crystallization

Silver tri­fluoro­methane­sulfonate (0.200 g, 0.778 mmol) was added to an aceto­nitrile solution of isonicotinamide (0.190 g, 1.56 mmol) and stirred for 30 min. The resulting clear solution was used to grow crystals by vapor diffusion with diethyl ether at 278 K.

## Refinement

Crystal data, data collection and structure refinement details are summarized in Table 3 ▸.

## Supplementary Material

Crystal structure: contains datablock(s) I. DOI: 10.1107/S2414314621010737/bx4019sup1.cif

Structure factors: contains datablock(s) I. DOI: 10.1107/S2414314621010737/bx4019Isup2.hkl

Supporting information file. DOI: 10.1107/S2414314621010737/bx4019Isup3.mol

CCDC reference: 2116012

Additional supporting information:  crystallographic information; 3D view; checkCIF report

## Figures and Tables

**Table 1:** Selected geometric parameters (Å, °)

Ag1—N3	2.162 (2)	Ag1—N1	2.162 (2)
			
N1—Ag1—N3	172.78 (7)	N2—C6—C3	117.3 (2)
N4—C12—C9	118.3 (2)		

**Table 2:** Hydrogen-bond geometry (Å, °)

*D*—H⋯*A*	*D*—H	H⋯*A*	*D*⋯*A*	*D*—H⋯*A*
N2—H2*A*⋯O2^i^	0.88	2.06	2.898 (3)	160
N2—H2*B*⋯O3	0.88	2.09	2.939 (3)	162
N4—H4*A*⋯O1^ii^	0.88	2.05	2.927 (3)	171
N4—H4*B*⋯O5^iii^	0.88	2.22	3.033 (3)	154

Symmetry codes: (i) 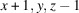 ; (ii) 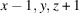 ; (iii) 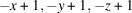 .

**Table 3:** Experimental details

Crystal data	
Chemical formula	[Ag(C_6_H_6_N_2_O)_2_](CF_3_O_3_S)·2C_2_H_3_N)
*M*_r_	583.30
Crystal system, space group	Triclinic, *P* 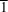
Temperature (K)	100
*a*, *b*, *c* (Å)	9.4566 (2), 11.0330 (3), 11.9848 (3)
α, β, γ (°)	114.000 (2), 103.9287 (19), 95.129 (2)
*V* (Å^3^)	1083.72 (5)
*Z*	2
Radiation type	Cu *K*α
μ (mm^−1^)	9.00
Crystal size (mm)	0.27 × 0.10 × 0.07

Data collection	
Diffractometer	XtaLAB Synergy, Dualflex, HyPix
Absorption correction	Gaussian (*CrysAlis PRO*; Rigaku OD, 2019 ▸)
*T*_min_, *T*_max_	0.544, 1.000
No. of measured, independent and observed [*I* > 2σ(*I*)] reflections	24470, 4387, 4211
*R*_int_	0.044
(sin θ/λ)_max_ (Å^−1^)	0.631

Refinement	
*R*[*F* ^2^ > 2σ(*F* ^2^)], *wR*(*F* ^2^), *S*	0.027, 0.072, 1.08
No. of reflections	4387
No. of parameters	300
H-atom treatment	H-atom parameters constrained
Δρ_max_, Δρ_min_ (e Å^−3^)	0.77, −0.78

Computer programs: *CrysAlis PRO* (Rigaku OD, 2019 ▸), *SHELXT* (Sheldrick, 2015*a* ▸), *SHELXL2018/3* (Sheldrick, 2015*b* ▸), and *OLEX2* (Dolomanov *et al.*, 2009 ▸).

## full crystallographic data

### Crystal data

**Table:**

[Ag(C_6_H_6_N_2_O)_2_](CF_3_O_3_S)·2C_2_H_3_N)	*Z* = 2
*M**_r_* = 583.30	*F*(000) = 584
Triclinic, *P*1	*D*_x_ = 1.788 Mg m^−^^3^
*a* = 9.4566 (2) Å	Cu *K*α radiation, λ = 1.54184 Å
*b* = 11.0330 (3) Å	Cell parameters from 12499 reflections
*c* = 11.9848 (3) Å	θ = 4.2–76.2°
α = 114.000 (2)°	µ = 9.00 mm^−^^1^
β = 103.9287 (19)°	*T* = 100 K
γ = 95.129 (2)°	Plate, clear colourless
*V* = 1083.72 (5) Å^3^	0.27 × 0.10 × 0.07 mm

### Data collection

**Table:**

XtaLAB Synergy, Dualflex, HyPix diffractometer	4387 independent reflections
Radiation source: micro-focus sealed X-ray tube, PhotonJet (Cu) X-ray Source	4211 reflections with *I* > 2σ(*I*)
Mirror monochromator	*R*_int_ = 0.044
Detector resolution: 10.0000 pixels mm^-1^	θ_max_ = 76.5°, θ_min_ = 4.2°
ω scans	*h* = −10→11
Absorption correction: gaussian (CrysAlisPro; Rigaku OD, 2019)	*k* = −13→13
*T*_min_ = 0.544, *T*_max_ = 1.000	*l* = −15→14
24470 measured reflections	

### Refinement

**Table:**

Refinement on *F*^2^	Primary atom site location: dual
Least-squares matrix: full	Hydrogen site location: inferred from neighbouring sites
*R*[*F*^2^ > 2σ(*F*^2^)] = 0.027	H-atom parameters constrained
*wR*(*F*^2^) = 0.072	*w* = 1/[σ^2^(*F*_o_^2^) + (0.0394*P*)^2^ + 0.7*P*] where *P* = (*F*_o_^2^ + 2*F*_c_^2^)/3
*S* = 1.08	(Δ/σ)_max_ = 0.002
4387 reflections	Δρ_max_ = 0.77 e Å^−^^3^
300 parameters	Δρ_min_ = −0.78 e Å^−^^3^
0 restraints	

### Special details

**Table:**

Geometry. All esds (except the esd in the dihedral angle between two l.s. planes) are estimated using the full covariance matrix. The cell esds are taken into account individually in the estimation of esds in distances, angles and torsion angles; correlations between esds in cell parameters are only used when they are defined by crystal symmetry. An approximate (isotropic) treatment of cell esds is used for estimating esds involving l.s. planes.

### Fractional atomic coordinates and isotropic or equivalent isotropic displacement parameters (Å^2^)

**Table:**

	*x*	*y*	*z*	*U*_iso_*/*U*_eq_	
Ag1	0.33026 (2)	0.42666 (2)	0.38452 (2)	0.01913 (7)	
S1	0.80813 (6)	0.92406 (6)	0.27008 (6)	0.01835 (13)	
F1	0.59457 (16)	1.01941 (15)	0.16879 (15)	0.0272 (3)	
F3	0.77762 (19)	1.00737 (17)	0.09034 (15)	0.0314 (4)	
F2	0.80186 (19)	1.16528 (15)	0.27750 (16)	0.0353 (4)	
O2	−0.13731 (19)	0.48669 (17)	0.77676 (16)	0.0195 (3)	
O1	0.68870 (19)	0.28645 (17)	−0.09989 (16)	0.0209 (4)	
O5	0.96843 (19)	0.96695 (17)	0.31236 (17)	0.0238 (4)	
O3	0.7525 (2)	0.79175 (17)	0.16192 (18)	0.0263 (4)	
N3	0.1996 (2)	0.40757 (19)	0.50408 (18)	0.0151 (4)	
N1	0.4587 (2)	0.4184 (2)	0.25573 (18)	0.0156 (4)	
O4	0.7400 (2)	0.9518 (2)	0.37019 (19)	0.0350 (5)	
N2	0.7732 (2)	0.5144 (2)	−0.0006 (2)	0.0191 (4)	
H2A	0.822879	0.511950	−0.054633	0.023*	
H2B	0.774705	0.592520	0.062122	0.023*	
N4	−0.1246 (2)	0.2695 (2)	0.73398 (19)	0.0184 (4)	
H4A	−0.184009	0.264893	0.779082	0.022*	
H4B	−0.088239	0.198602	0.695256	0.022*	
N6	0.1601 (2)	0.1602 (2)	0.1866 (2)	0.0262 (5)	
N5	0.4275 (3)	0.7251 (2)	0.5268 (2)	0.0325 (5)	
C9	0.0129 (2)	0.3874 (2)	0.6467 (2)	0.0150 (4)	
C3	0.6141 (2)	0.4105 (2)	0.0827 (2)	0.0149 (4)	
C2	0.5905 (3)	0.5331 (2)	0.1669 (2)	0.0164 (5)	
H2	0.627509	0.615873	0.166627	0.020*	
C10	0.0835 (2)	0.5137 (2)	0.6652 (2)	0.0150 (4)	
H10	0.068538	0.594775	0.726905	0.018*	
C8	0.0393 (3)	0.2713 (2)	0.5556 (2)	0.0167 (5)	
H8	−0.005429	0.183595	0.541580	0.020*	
C11	0.1752 (3)	0.5196 (2)	0.5931 (2)	0.0157 (4)	
H11	0.223022	0.606106	0.606713	0.019*	
C12	−0.0895 (3)	0.3837 (2)	0.7243 (2)	0.0158 (4)	
C5	0.4811 (3)	0.3005 (2)	0.1740 (2)	0.0174 (5)	
H5	0.443286	0.219160	0.176380	0.021*	
C6	0.6954 (3)	0.3993 (2)	−0.0140 (2)	0.0165 (5)	
C1	0.5128 (3)	0.5324 (2)	0.2506 (2)	0.0171 (5)	
H1	0.496800	0.616240	0.307082	0.021*	
C4	0.5567 (3)	0.2924 (2)	0.0869 (2)	0.0165 (5)	
H4	0.569408	0.206974	0.030278	0.020*	
C7	0.1313 (3)	0.2857 (2)	0.4862 (2)	0.0166 (5)	
H7	0.147119	0.206188	0.423132	0.020*	
C16	0.0818 (3)	0.1753 (2)	0.1065 (2)	0.0220 (5)	
C17	−0.0166 (3)	0.1959 (3)	0.0045 (3)	0.0261 (5)	
H17A	−0.119652	0.152936	−0.012235	0.039*	
H17B	0.012716	0.155320	−0.073582	0.039*	
H17C	−0.009032	0.293417	0.030980	0.039*	
C14	0.5027 (3)	0.8301 (3)	0.5709 (3)	0.0259 (6)	
C13	0.7428 (3)	1.0349 (2)	0.1983 (2)	0.0213 (5)	
C15	0.6018 (3)	0.9632 (3)	0.6278 (3)	0.0302 (6)	
H15A	0.642520	0.973096	0.563323	0.036*	
H15B	0.683648	0.971724	0.700319	0.036*	
H15C	0.545821	1.034243	0.657809	0.036*	

### Atomic displacement parameters (Å^2^)

**Table:**

	*U*^11^	*U*^22^	*U*^33^	*U*^12^	*U*^13^	*U*^23^
Ag1	0.02101 (11)	0.02377 (11)	0.01987 (11)	0.00738 (7)	0.01347 (7)	0.01206 (8)
S1	0.0220 (3)	0.0175 (3)	0.0209 (3)	0.0087 (2)	0.0108 (2)	0.0103 (2)
F1	0.0224 (7)	0.0293 (8)	0.0322 (8)	0.0106 (6)	0.0075 (6)	0.0153 (7)
F3	0.0405 (9)	0.0372 (9)	0.0328 (9)	0.0161 (7)	0.0204 (7)	0.0243 (7)
F2	0.0379 (9)	0.0142 (7)	0.0423 (10)	0.0057 (6)	0.0000 (8)	0.0083 (7)
O2	0.0237 (9)	0.0203 (8)	0.0220 (8)	0.0116 (7)	0.0139 (7)	0.0113 (7)
O1	0.0266 (9)	0.0191 (8)	0.0220 (9)	0.0069 (7)	0.0151 (7)	0.0093 (7)
O5	0.0220 (9)	0.0198 (8)	0.0302 (10)	0.0066 (7)	0.0069 (7)	0.0117 (7)
O3	0.0290 (10)	0.0154 (8)	0.0335 (10)	0.0036 (7)	0.0094 (8)	0.0100 (8)
N3	0.0163 (9)	0.0182 (9)	0.0141 (9)	0.0071 (7)	0.0076 (7)	0.0080 (8)
N1	0.0157 (9)	0.0194 (9)	0.0153 (9)	0.0062 (7)	0.0073 (8)	0.0092 (8)
O4	0.0402 (12)	0.0530 (13)	0.0336 (11)	0.0270 (10)	0.0243 (9)	0.0294 (10)
N2	0.0229 (10)	0.0198 (10)	0.0190 (10)	0.0041 (8)	0.0137 (8)	0.0089 (8)
N4	0.0220 (10)	0.0187 (10)	0.0229 (10)	0.0086 (8)	0.0154 (8)	0.0116 (8)
N6	0.0259 (11)	0.0295 (11)	0.0251 (11)	0.0045 (9)	0.0108 (9)	0.0125 (10)
N5	0.0456 (15)	0.0285 (13)	0.0290 (12)	0.0141 (11)	0.0177 (11)	0.0133 (10)
C9	0.0143 (11)	0.0192 (11)	0.0139 (11)	0.0049 (9)	0.0044 (9)	0.0093 (9)
C3	0.0132 (10)	0.0173 (11)	0.0165 (11)	0.0050 (8)	0.0047 (9)	0.0092 (9)
C2	0.0174 (11)	0.0175 (11)	0.0178 (11)	0.0058 (9)	0.0069 (9)	0.0098 (9)
C10	0.0163 (11)	0.0156 (10)	0.0135 (10)	0.0061 (8)	0.0054 (9)	0.0058 (9)
C8	0.0178 (11)	0.0167 (11)	0.0184 (11)	0.0047 (9)	0.0075 (9)	0.0091 (9)
C11	0.0161 (11)	0.0164 (11)	0.0164 (11)	0.0052 (8)	0.0053 (9)	0.0085 (9)
C12	0.0151 (11)	0.0198 (11)	0.0147 (11)	0.0057 (9)	0.0055 (9)	0.0088 (9)
C5	0.0175 (11)	0.0171 (11)	0.0200 (12)	0.0040 (9)	0.0074 (9)	0.0094 (9)
C6	0.0143 (10)	0.0207 (11)	0.0180 (11)	0.0068 (9)	0.0057 (9)	0.0107 (9)
C1	0.0184 (11)	0.0183 (11)	0.0174 (11)	0.0060 (9)	0.0072 (9)	0.0091 (9)
C4	0.0177 (11)	0.0169 (11)	0.0162 (11)	0.0054 (9)	0.0074 (9)	0.0070 (9)
C7	0.0201 (11)	0.0161 (11)	0.0158 (11)	0.0059 (9)	0.0082 (9)	0.0072 (9)
C16	0.0253 (13)	0.0204 (11)	0.0227 (13)	0.0038 (10)	0.0143 (11)	0.0081 (10)
C17	0.0293 (14)	0.0265 (13)	0.0261 (13)	0.0082 (11)	0.0116 (11)	0.0131 (11)
C14	0.0328 (14)	0.0335 (15)	0.0226 (13)	0.0188 (12)	0.0154 (11)	0.0170 (12)
C13	0.0243 (12)	0.0169 (11)	0.0247 (13)	0.0077 (9)	0.0091 (10)	0.0095 (10)
C15	0.0310 (15)	0.0315 (14)	0.0327 (15)	0.0113 (12)	0.0126 (12)	0.0162 (12)

### Geometric parameters (Å, º)

**Table:**

Ag1—N3	2.162 (2)	C9—C12	1.505 (3)
Ag1—N1	2.162 (2)	C3—C2	1.396 (3)
S1—O5	1.4442 (18)	C3—C6	1.510 (3)
S1—O3	1.4433 (18)	C3—C4	1.390 (3)
S1—O4	1.434 (2)	C2—H2	0.9500
S1—C13	1.831 (2)	C2—C1	1.380 (3)
F1—C13	1.337 (3)	C10—H10	0.9500
F3—C13	1.333 (3)	C10—C11	1.378 (3)
F2—C13	1.331 (3)	C8—H8	0.9500
O2—C12	1.240 (3)	C8—C7	1.380 (3)
O1—C6	1.235 (3)	C11—H11	0.9500
N3—C11	1.348 (3)	C5—H5	0.9500
N3—C7	1.347 (3)	C5—C4	1.380 (3)
N1—C5	1.346 (3)	C1—H1	0.9500
N1—C1	1.345 (3)	C4—H4	0.9500
N2—H2A	0.8800	C7—H7	0.9500
N2—H2B	0.8800	C16—C17	1.459 (4)
N2—C6	1.338 (3)	C17—H17A	0.9800
N4—H4A	0.8800	C17—H17B	0.9800
N4—H4B	0.8800	C17—H17C	0.9800
N4—C12	1.331 (3)	C14—C15	1.460 (4)
N6—C16	1.144 (3)	C15—H15A	0.9800
N5—C14	1.141 (4)	C15—H15B	0.9800
C9—C10	1.395 (3)	C15—H15C	0.9800
C9—C8	1.395 (3)		
			
N1—Ag1—N3	172.78 (7)	O2—C12—C9	118.8 (2)
O5—S1—C13	103.79 (11)	N4—C12—C9	118.3 (2)
O3—S1—O5	113.68 (11)	N1—C5—H5	118.5
O3—S1—C13	101.70 (11)	N1—C5—C4	122.9 (2)
O4—S1—O5	115.49 (12)	C4—C5—H5	118.5
O4—S1—O3	116.01 (13)	O1—C6—N2	123.2 (2)
O4—S1—C13	103.62 (11)	O1—C6—C3	119.5 (2)
C11—N3—Ag1	119.93 (16)	N2—C6—C3	117.3 (2)
C7—N3—Ag1	121.89 (15)	N1—C1—C2	123.0 (2)
C7—N3—C11	118.0 (2)	N1—C1—H1	118.5
C5—N1—Ag1	122.04 (16)	C2—C1—H1	118.5
C1—N1—Ag1	120.28 (16)	C3—C4—H4	120.3
C1—N1—C5	117.6 (2)	C5—C4—C3	119.4 (2)
H2A—N2—H2B	120.0	C5—C4—H4	120.3
C6—N2—H2A	120.0	N3—C7—C8	122.9 (2)
C6—N2—H2B	120.0	N3—C7—H7	118.5
H4A—N4—H4B	120.0	C8—C7—H7	118.5
C12—N4—H4A	120.0	N6—C16—C17	179.3 (3)
C12—N4—H4B	120.0	C16—C17—H17A	109.5
C10—C9—C12	118.3 (2)	C16—C17—H17B	109.5
C8—C9—C10	118.2 (2)	C16—C17—H17C	109.5
C8—C9—C12	123.5 (2)	H17A—C17—H17B	109.5
C2—C3—C6	123.5 (2)	H17A—C17—H17C	109.5
C4—C3—C2	117.9 (2)	H17B—C17—H17C	109.5
C4—C3—C6	118.5 (2)	N5—C14—C15	178.8 (3)
C3—C2—H2	120.5	F1—C13—S1	110.99 (17)
C1—C2—C3	119.1 (2)	F3—C13—S1	111.58 (16)
C1—C2—H2	120.5	F3—C13—F1	107.2 (2)
C9—C10—H10	120.3	F2—C13—S1	111.75 (17)
C11—C10—C9	119.4 (2)	F2—C13—F1	107.49 (19)
C11—C10—H10	120.3	F2—C13—F3	107.6 (2)
C9—C8—H8	120.5	C14—C15—H15A	109.5
C7—C8—C9	118.9 (2)	C14—C15—H15B	109.5
C7—C8—H8	120.5	C14—C15—H15C	109.5
N3—C11—C10	122.6 (2)	H15A—C15—H15B	109.5
N3—C11—H11	118.7	H15A—C15—H15C	109.5
C10—C11—H11	118.7	H15B—C15—H15C	109.5
O2—C12—N4	122.8 (2)		
			
Ag1—N3—C11—C10	174.85 (16)	C2—C3—C4—C5	−0.8 (3)
Ag1—N3—C7—C8	−175.54 (17)	C10—C9—C8—C7	−1.2 (3)
Ag1—N1—C5—C4	176.85 (17)	C10—C9—C12—O2	17.8 (3)
Ag1—N1—C1—C2	−177.40 (17)	C10—C9—C12—N4	−162.0 (2)
O5—S1—C13—F1	−172.06 (16)	C8—C9—C10—C11	0.4 (3)
O5—S1—C13—F3	68.43 (19)	C8—C9—C12—O2	−161.1 (2)
O5—S1—C13—F2	−52.1 (2)	C8—C9—C12—N4	19.1 (3)
O3—S1—C13—F1	69.70 (18)	C11—N3—C7—C8	−0.6 (3)
O3—S1—C13—F3	−49.8 (2)	C12—C9—C10—C11	−178.5 (2)
O3—S1—C13—F2	−170.33 (18)	C12—C9—C8—C7	177.7 (2)
N1—C5—C4—C3	0.6 (3)	C5—N1—C1—C2	−0.7 (3)
O4—S1—C13—F1	−51.0 (2)	C6—C3—C2—C1	179.0 (2)
O4—S1—C13—F3	−170.54 (18)	C6—C3—C4—C5	−179.5 (2)
O4—S1—C13—F2	68.9 (2)	C1—N1—C5—C4	0.2 (3)
C9—C10—C11—N3	0.3 (3)	C4—C3—C2—C1	0.4 (3)
C9—C8—C7—N3	1.3 (3)	C4—C3—C6—O1	11.6 (3)
C3—C2—C1—N1	0.4 (3)	C4—C3—C6—N2	−167.8 (2)
C2—C3—C6—O1	−167.0 (2)	C7—N3—C11—C10	−0.2 (3)
C2—C3—C6—N2	13.6 (3)		

### Hydrogen-bond geometry (Å, º)

**Table:**

*D*—H···*A*	*D*—H	H···*A*	*D*···*A*	*D*—H···*A*
N2—H2*A*···O2^i^	0.88	2.06	2.898 (3)	160
N2—H2*B*···O3	0.88	2.09	2.939 (3)	162
N4—H4*A*···O1^ii^	0.88	2.05	2.927 (3)	171
N4—H4*B*···O5^iii^	0.88	2.22	3.033 (3)	154

Symmetry codes: (i) *x*+1, *y*, *z*−1; (ii) *x*−1, *y*, *z*+1; (iii) −*x*+1, −*y*+1, −*z*+1.
